# Surgery for pseudoaneurysm of the ascending aorta under moderate hypothermia

**DOI:** 10.1186/1749-8090-6-125

**Published:** 2011-09-30

**Authors:** Tae-Eun Jung, Dong-Hyup Lee

**Affiliations:** 1 Department of Thoracic and Cardiovascular Surgery, College of Medicine, Yeungnam University, Daegu, Korea

**Keywords:** aortic pseudoaneurysm, aortic valve replacement, moderate hypothermia

## Abstract

Pseudoaneurysm of the ascending aorta is a rare complication after cardiac surgery. Particularly, pseudoaneurysm due to postoperative infection in the ascending aorta requires surgical treatment with antibiotics. If a large sized pseudoaneurysm is located at the retrosternal space, then there is a very high risk of massive bleeding from rupture during performance of resternotomy. To avoid this risk, we performed femoro-femoral bypass under moderate hypothermia with transient circulatory arrest, and we report here on the successful result of this case.

## Background

Thoracic aortic pseudoaneurysm is a very rare complication after cardiac surgery with an incidence of less than 0.5% [1]. It has been reported that leaking at an aortic cannulation site is the major risk factor of pseudoaneurysm [2], and deep sternal infection or an increased possibility of suture dehiscence, such as after an ascending aortic dissection, showed the high occurrence of pseudoaneurysm [3]. A simple chest PA can detect widening of the mediastinum if the pseudoaneurysm is large and the diagnosis can be confirmed with chest CT and an echocardiogram.

## Case presentation

A 69 years old female patient was hospitalized for dyspnea, which was her chief complaint. The echocardiogram during the visit showed severe mitral stenosis (MVA = 0.8 cm^2^) and aortic stenosis (AVA = 0.7 cm^2^). Mild pulmonary hypertension (RVSP = 34 mmHg) and grade I tricuspid regurgitation were also present with a left ventricular ejection fraction of 43%. Left ventricular hypertrophy was present and the left ventricular wall motion was generalized hypokinetic, but no localized wall motion abnormality was found. According to the coronary artery angiogram, there was no stenosis of the coronary artery and the patient had no other significant medical history except for treatment for hypertension.

During the surgery, the aortic valve and mitral valve were replaced with Hancock^® ^II (Medtronic) 23 mm and Hancock II (Medtronic) 27 mm, respectively, and then no postoperative complications were observed.

**Figure 1:**
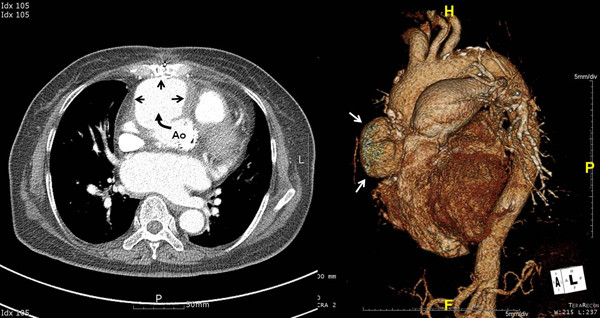
**The preoperative chest CT scan shows a large pseudoaneurysm in the ascending aorta (black and white arrows)**.

**Figure 2:**
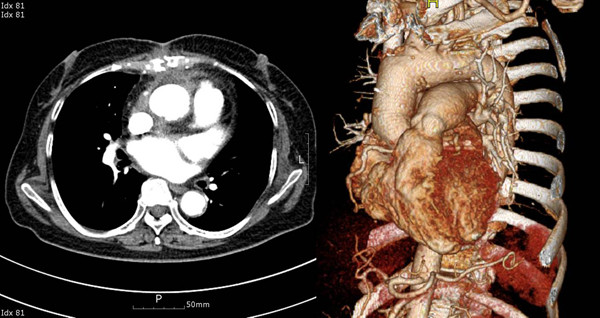
**The Postoperative chest CT scan shows the ascending aortic graft without pseudoaneurysm**.

On the 8^th ^postoperative day, sternal infection was noted and *pseudomonas aeruginosa *was cultured on the culture test. The patient experienced only mild fever, so wound care and ceftazidime IV were concurrently administered. Curettage and debridement of the infected sternum were scheduled and we continued observing the patient. On the 11^th ^postoperative day, there was massive bleeding at the retrosternal area, so an emergency operation was performed. The bleeding site was around the aortic vent insertion site and the aortic adventitia was very weak due to infection. The weakened ascending aorta tissue was removed and patch repair was performed using a Hemashield^® ^graft (Boston Scientific). She was continually given antibiotic medication for six weeks and there was no abnormal finding on the echocardiogram before discharge from the hospital. Two weeks after the discharge, the patient was hospitalized again from a 38°C fever and chills, and *pseudomonas aeruginosa *was cultured from her blood. Chest CT confirmed a large sized ascending aortic pseudoaneurysm (Figure 1). The pseudoaneurysm's largest diameter was 7 cm and its anterior surface was adhered to the retrosternal region. Considering the size and location of the pseudoaneurysm, the surgery approach had a very high risk of massive bleeding. However, there was an aorta clamping site, so instead of deep hypothermia, moderate hypothermia was planned to lower the body temperature for sternotomy. Still, rupture of the aortic pseudoaneurysm was inevitable. Hence, after transient circulatory arrest, aortic cross-clamping while maintaining cardiopulmonary bypass (CPB) to perform graft interposition was planned. First, the right femoral artery and vein were each cannulated. Then, CPB was carried out and sternotomy was performed when the body temperature reached 25°C. Immediately after the sternotomy, the pseudoaneurysm ruptured and caused massive bleeding, so circulatory arrest was followed by cross-clamping at the distal ascending aorta. The CPB was restarted after 3 minutes. HTK solution was directly injected to the coronary artery for cardioplegia. The previous patch repair on the ascending aorta tissue was removed because of the infection, and then a Hemashield graft (Boston Scientific) 30 mm was used to perform ascending aorta graft interposition. The total aortic cross-clamp time was 121 minutes. Atrial fibrillation occurred after the surgery, but the vital signs remained safe, so the ventilator tube was removed on the 5^th ^postoperative day. There was no postoperative neurologic complication, the antibiotics medication was continued for 9 weeks and repeated blood cultures showed no bacterial growth, so she was discharged from the hospital. On the 90^th ^postoperative day, chest CT did not show leaking at the graft anastomosis site of the ascending aorta (Figure 2).

## Discussion

With its high risk of rupture, a pseudoaneurysm requires an immediate operation when diagnosed due to the high mortality rate. Despite the recent reports of percutaneously excluding false aneurysms, surgery is still necessary for most cases [4]. An ascending aortic pseudoaneurysm has high morbidity and the mortality rate has ranged from 29%-46% in the medical literature [5], and this is due to fatal bleeding from rupture of a pseudoaneurysm upon sternal reentry [2]. If a pseudoaneurysm is large and it is located anteriorly and it has eroded into the outer sternum, then there is a very high risk of massive hemorrhage during the surgery [6]. The ascending aortic pseudoaneurysm mainly bulges anteriorly while eroding the boney structures of the sternum. Although rarely reported, ascending aortic pseudoaneurysm can occur posteriorly from an injury caused by a cardioplegia cannula [7].

The most important part of the surgery is to avoid bleeding during resternotomy and to maintain proper cerebral perfusion [6]. Before resternotomy, carotid artery cannulation is performed for CPB, but femoro-femoral bypass and deep hypothermia have been widely used with satisfactory results [1]. Although the cannulation method can vary according to the size and the location of the ascending aortic pseudoaneurysm, femoro-femoral bypass and deep hypothermic circulatory arrest have high variability for the circulatory arrest time depending on the severity of adhesion, the size of the pseudoaneurysm and the size of the leak [8].

We think that various methods will continue to be tried to reduce the neurologic complications following circulatory arrest. For this case, aortic cross-clamping was determined to be possible on the distal ascending aorta; subsequently, ascending aorta graft interposition was performed under moderate hypothermia with transient circulatory arrest.

Edwin *et al *[9] emphasized ways to prevent postoperative pseudoaneurysm with performing proper suture technique, careful handling of the aorta wall, strict asepsis and aggressive treatment of perioperative infection.

## Conclusions

If a large sized pseudoaneurysm is located at the retrosternal space, then there is a very high risk of massive bleeding from rupture during performance of resternotomy. To avoid this risk, we performed femoro-femoral bypass under moderate hypothermia with transient circulatory arrest, and we report here on the successful result of this case.

## Consent

Written informed consent was obtained from the patient for publication of this case report and accompanying images. A copy of the written consent is available for review by the Editor-in-Chief of this journal.

## Competing interests

The authors declare that they have no competing interests.

## Authors' contributions

TJ wrote the draft of the manuscript and obtained the written consent. DL performed the literature review and participated in the manuscript writing and helped to the final writing of the paper and gave final approval of the manuscript. All authors have read and approved the final manuscript.
